# Supplementary material for: Deep learning explains the biology of branched glycans from single-cell sequencing data
Source: iScience. 2022 Sep 19;25(10):105163. doi: 10.1016/j.isci.2022.105163 (PMC9547197; doi:10.1016/j.isci.2022.105163)
Supplement: Document S1. Figures S1–S4 [file mmc1.pdf]

iScience, Volume *25*

Supplemental information

Deep learning explains the biology of branched glycans from single-cell sequencing data

Rui Qin, Lara K. Mahal, and Daniel Bojar

Supplemental Figures

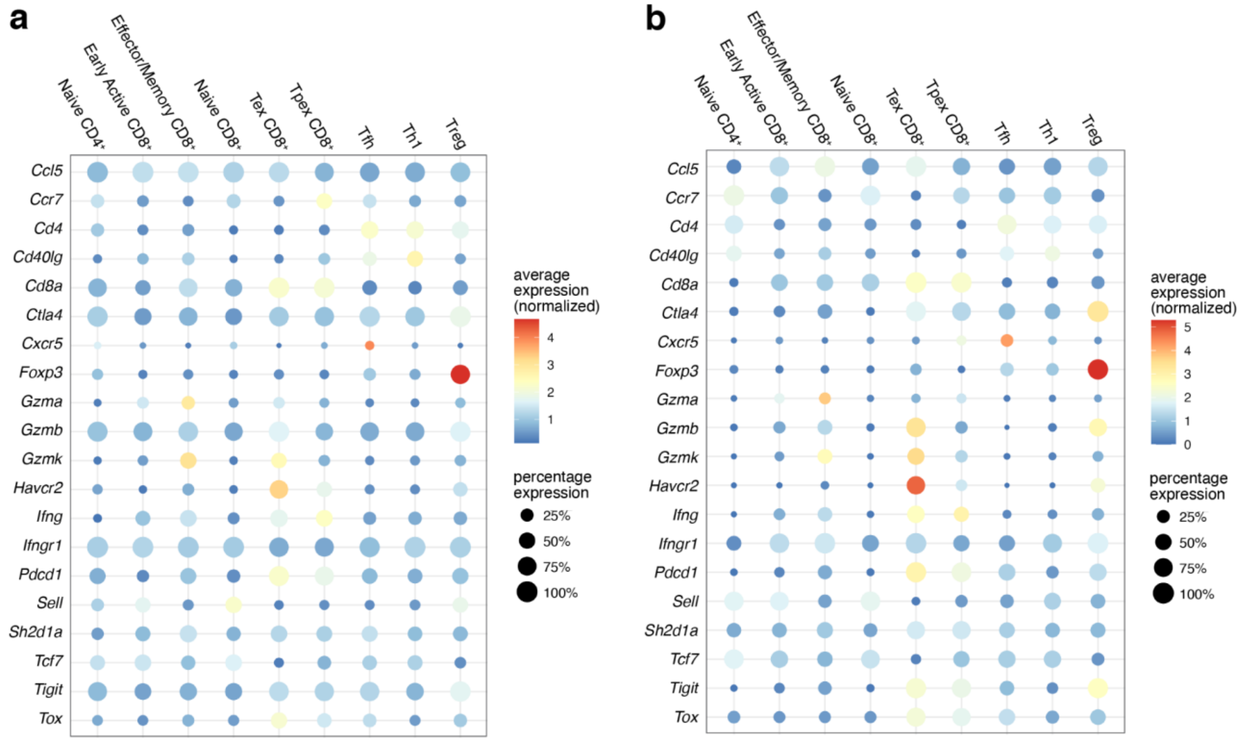

Figure S1. Expression of marker genes of subtypes of T cells in the (**a**) TIL dataset and (**b**) LN dataset, Related to Figure 1. For each gene, the average expression has been normalized to the mean of expression of the gene in all types of cells.

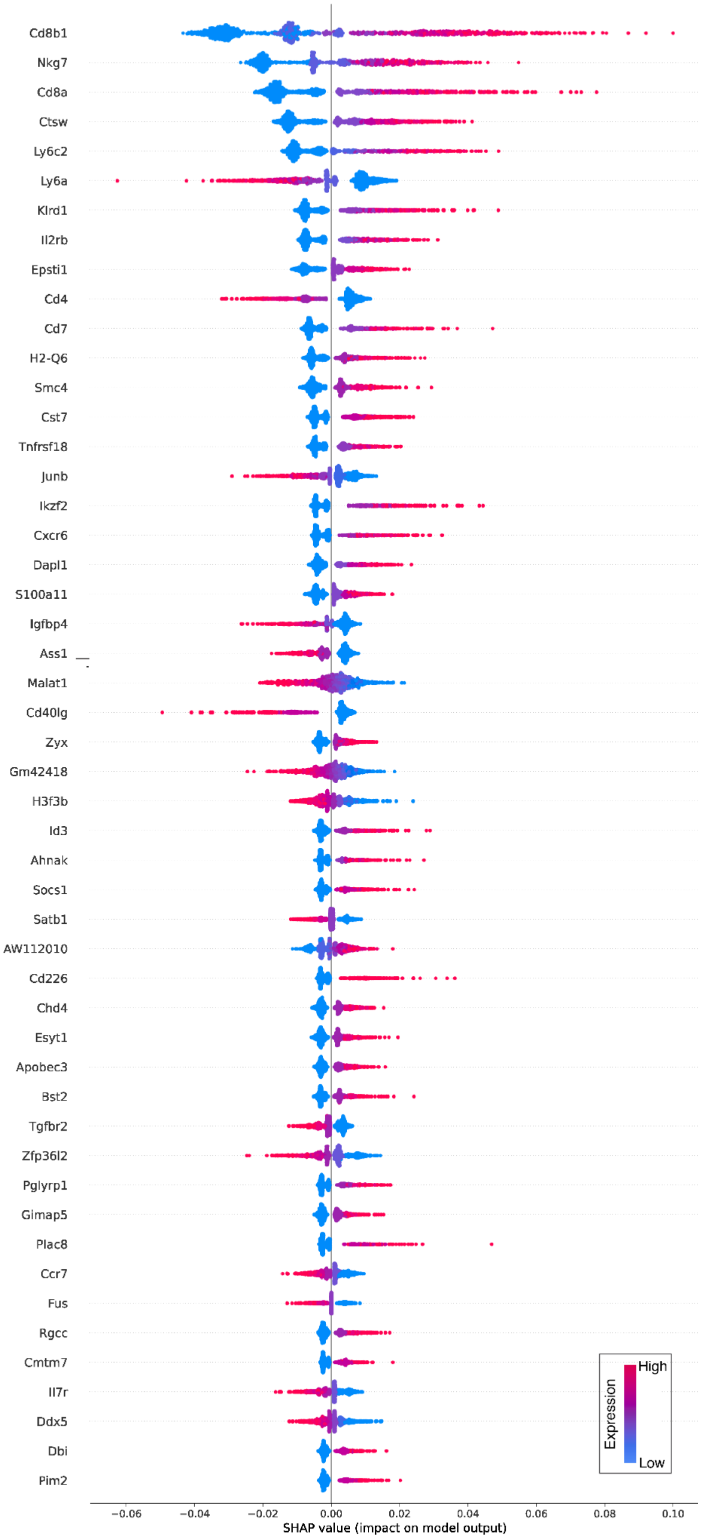

Figure S2. SHAP values of top 50 genes in the LN dataset ranked by median absolute SHAP value, Related to Figure 3.

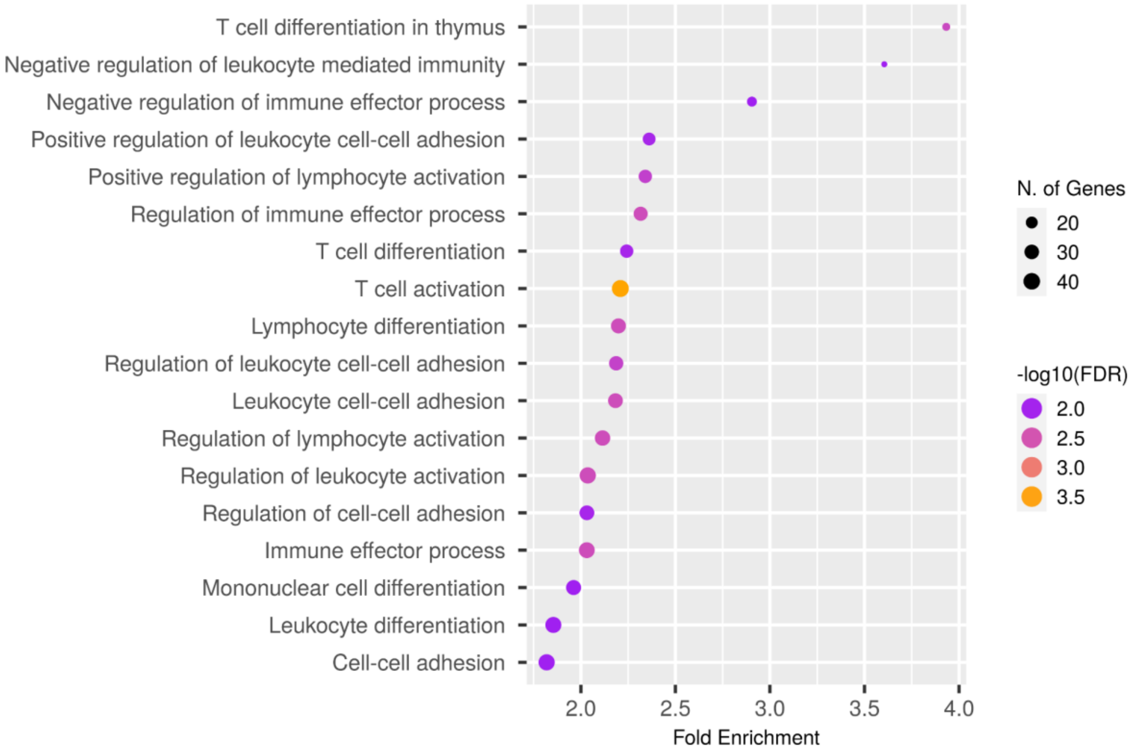

Figure S3. Gene Ontology pathway enrichment analysis of using the SHAP genes of the LN dataset, Related to Figure 3.

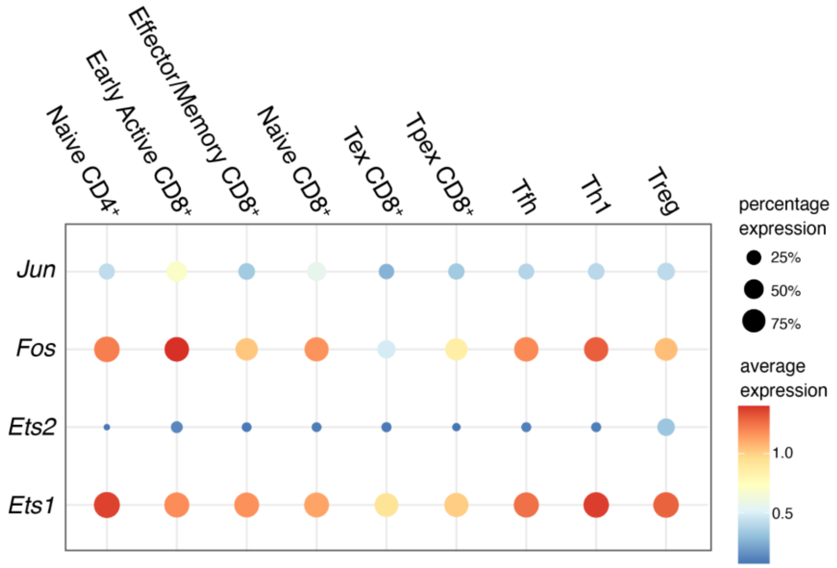

Figure S4. Expression of four genes encoding transcription factors regulating MGAT5 expression across subtypes of T cells in the TIL dataset, Related to Figure 5.
